# Characterization of Fumonisin A-Series by High-Resolution Liquid Chromatography-Orbitrap Mass Spectrometry

**DOI:** 10.3390/toxins6082580

**Published:** 2014-08-21

**Authors:** Masayoshi Tamura, Naoki Mochizuki, Yasushi Nagatomi, Akira Toriba, Kazuichi Hayakawa

**Affiliations:** 1 Research Laboratories for Food Safety Chemistry, Asahi Group Holdings, Ltd., 1-21, Midori 1-chome, Moriya-shi, Ibaraki 302-0106, Japan; E-Mails: masayoshi.tamura@asahigroup-holdings.com (M.T.); yasushi.nagatomi@asahigroup-holdings.com (Y.N.); 2 Graduate School of Medical Sciences, Kanazawa University, Kakuma-machi, Kanazawa-shi, Kanazawa 920-1192, Japan; 3 Institute of Medical, Pharmaceutical and Health Sciences, Kanazawa University, Kakuma-machi, Kanazawa-shi, Kanazawa 920-1192, Japan; E-Mails: toriba@p.kanazawa-u.ac.jp (A.T.); hayakawa@p.kanazawa-u.ac.jp (K.H.)

**Keywords:** LC-Orbitrap MS, fumonisin A, corn

## Abstract

Fumonisin A-series (FAs) in a reference material of corn sample that was naturally contaminated with fumonisins was characterized using high-resolution liquid chromatography-Orbitrap mass spectrometry (LC-Orbitap MS). Peaks for fumonisin B1 (FB1), fumonisin B2 (FB2), and fumonisin B3 (FB3), in addition to three peaks corresponding to unknown compounds I, II, and III, were detected in the chromatogram for the corn sample. Fragment ion analysis for FB1, FB2, and FB3 showed that while the ions formed at *m*/*z* values of 200–800 were similar to those formed by the cleavage of the tricarballylic acids and the hydroxyl groups, the fragmentation patterns at *m*/*z* values of 50–200 varied depending on the hydroxyl group locations in the compounds. Fragment ion analysis of compounds I–III revealed structural similarities to FBs, only differing by an additional C_2_H_2_O in the unknown compounds. Using these results and by comparing the product ion mass spectra of compound I with fumonisin A1 (FA1) synthesized from FB1 standards, compounds I–III were hypothesized to be *N*-acetyl analogs of FBs: fumonisins A1 (FA1), A2 (FA2), and A3 (FA3). The method for determining concentrations was validated with FA1, FB1, FB2, and FB3 standards and applied to analyze the reference material. The FB1, FB2, and FB3 analytical levels were within acceptance limits and the amount of FA1 in the material was ~15% of FB1 amount at 4.2 mg/kg.

## 1. Introduction

**Figure 1:**
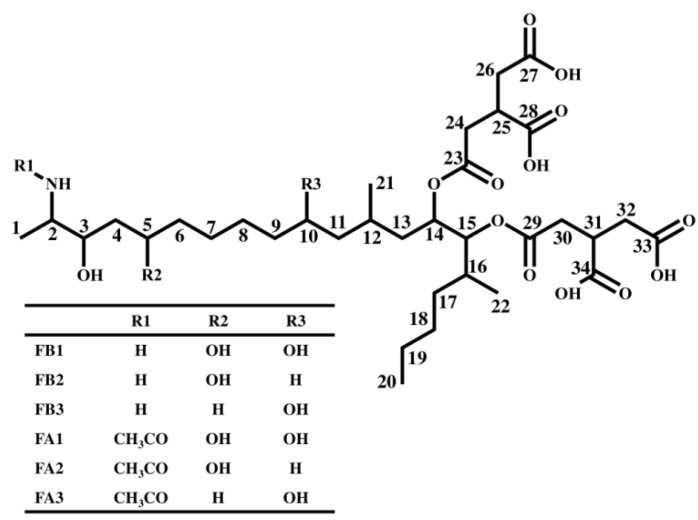
Chemical structure of fumonisins.

There is significant worldwide concern over the health risks posed by the presence of fumonisins (chemical structure shown in Figure 1), which are one of the key mycotoxin contaminants in corn. In particular, fumonisin B1 (FB1) causes equine leukoencephalomalacia, porcine pulmonary edema, and induces esophageal cancer in humans [1,2]. The fumonisin B-series (FBs) is known to inhibit sphingosine *N*-acyltransferase (ceramide synthase) in sphingolipid metabolism, which is important for the maintenance of membranes in all eukaryotic cells [1,2,3]. Additionally, FB1 is classified as 2B (*possibly carcinogenic to humans*) by the International Agency for Research on Cancer (IARC) [3]. Since the natural occurrence of FBs including FB1 have been reported worldwide, maximum levels for FBs in corn and corn products have been established in several countries including the USA and the EU [4,5]. Additionally, the Codex Alimentarius Commission has also advanced debates on establishing regulatory limits. There is a great desire for further research in the area of FBs, since there is much unknown information regarding them, such as the causal relationship between the action mechanisms of fumonisin and the onset of disease.

In addition to FBs, several analogs of this group including fumonisin A-series (*N-*acetyl analogs, FAs), the fumonisin C-series (demethyl analogs, FCs), and the fumonisin P-series (*N*-3-hydroxypiridinium analogs, FPs) have been discovered in the culture media of genus *Fusarium*. They are produced by *Fusarium moniliforme*, *F. verticillioides*, *F. proliferatum*, *F. nygami*, and *F. oxysporum* [6,7,8,9,10,11]. Similar to FBs, there are toxicity reports suggesting that FAs also have the ability to inhibit sphingosine *N*-acyltransferase [12]. In addition, FCs and FPs are known to be phytotoxic and cytotoxic [13]. Since there have been few reports of the detection of the fumonisin analogs in foods and feeds directly ingested by humans and animals, the extent to which they pose toxicity and contamination risks to cereals is unclear.

Recently, liquid chromatography (LC) coupled with triple-quadrupole mass spectrometry has been widely used for the quantification of mycotoxins including fumonisins in foods [14]. The triple-quadrupole mass spectrometry enables highly sensitive and selective detection of targeted compounds, although it is less suitable for a comprehensive determination since the analysis is performed by eliminating non-targeted compounds. In order to comprehensively analyze the mycotoxins, the LC-Orbitrap mass spectrometer (MS) has been introduced for the simultaneous screening [15,16] and characterization of mycotoxins [17,18,19], which enables comprehensive analysis with high selectivity by providing high resolution and enables exact mass measurements for a full mass scan. Further, this technique allows the estimation of the formulae of fragment ions by incorporating a quadrupole mass filter and collision cell in the Orbitrap.

In the present study, we have attempted to detect fumonisins analogs in commercially available reference material of corn sample (MTC-9999E) that was naturally contaminated with fumonisins using Q-Exactive, which is Orbitrap MS equipped with a quadrupole mass filter and a collision cell. We have also attempted to deduce the structure of the compounds detected in the product ion mass spectra, by performing a fragment analysis. Additionally, a technique to determine the amount of FB1, FB2, FB3, and fumonisin A1 (FA1) has been developed and applied for the quantification of FA1 in MTC-9999E.

## 2. Results and Discussion

### 2.1. Determination of Fumonisins by an LC-Orbitrap

**Figure 2:**
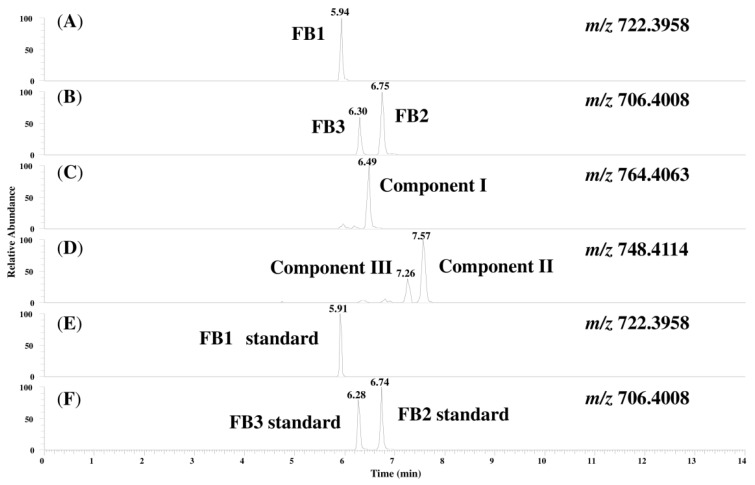
Chromatograms of the compounds in MTC-9999E and the standard solutions of FB1, FB2, and FB3: (**A**) FB1 in the sample; (**B**) FB2 and FB3 in the sample; (**C**) compound I; (**D**) compounds II and III; (**E**) FB1 standard; and (**F**) FB2 and FB3 standards.

Figure 2 shows chromatograms of the prepared MTC-9999E obtained with a full mass scan. The retention time for the FB1, FB2, and FB3 peaks detected from the corn sample were quite similar to those obtained for standard solutions. The peaks for FB1, FB2, and FB3 and three unknown compounds (hereafter referred to as compounds I, II, and III) were simultaneously detected and the measured mass, the theoretical mass, the mass error, and the calculated formulae are shown in Table 1. As seen in the table, the only difference between the calculated formulae of compounds I, II, and III and FB1, FB2, and FB3 was that the former contained an additional C_2_H_2_O compared to the latter. This suggests that compounds I, II, and III are likely to be FA1, fumonisin A2 (FA2), and fumonisin A3 (FA3), which are *N*-acetyl analogs of FB1, FB2, and FB3, respectively. Therefore, the product ion spectra for each peak were measured, and the fragment ions were structurally characterized, as described next.

**Table 1:** Characteristic peak for FB1, FB2, FB3, and unknown compounds in MTC-9999E.

Factor	FB1	FB2	FB3	Compound I	Compound II	Compound III
Measured mass (*m*/*z*)	722.3973	706.4020	706.4015	764.4059	748.4123	748.4118
Calculated formula	C_34_H_60_NO_15_^+^	C_34_H_60_NO_14_^+^	C_34_H_60_NO_14_^+^	C_36_H_62_NO_16_^+^	C_36_H_62_NO_15_^+^	C_36_H_62_NO_15_^+^
Theoretical mass (*m*/*z*)	722.3958	706.4008	706.4008	764.4063	748.4114	748.4114
Mass error (ppm)	1.59	1.21	0.66	−0.48	1.20	0.54

### 2.2. Characterization of the Fragment Ions for FB1, FB2, and FB3

**Figure 3:**
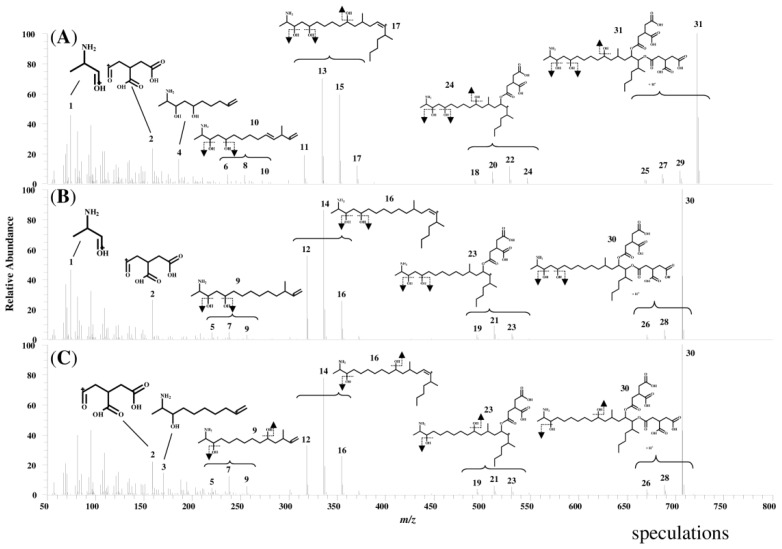
Product ion mass spectra of standard solutions of FB1, FB2, and FB3 (**A**) FB1 standard; (**B**) FB2 standard; and (**C**) FB3 standard.

Figure 3 shows product ion mass spectra of FB1, FB2, and FB3 standard solutions. The peaks in the spectra are labeled with identification (ID) numbers, corresponding to the numbers in Table 2, which summarizes the measured mass, the calculated formula, and the mass error for each spectrum. The product ion mass spectra of FB1, FB2, and FB3 in the corn sample were similar to those of the standard solutions.

**Table 2:** Characteristic peak assignment of the product ion mass spectra of FB1, FB2, and FB3 standard solutions.

ID	FB1			FB2			FB3		
	Measured Mass (*m*/*z*)	Calculated Formula [M + H]^+^	Mass Error (ppm)	Measured Mass (*m*/*z*)	Calculated Formula [M + H]^+^	Mass Error (ppm)	Measured Mass (*m*/*z*)	Calculated Formula [M + H]^+^	Mass Error (ppm)
1	74.0601	C_3_H_8_NO	1.17	74.0601	C_3_H_8_NO	1.17			
2	159.0290	C_6_H_7_O_5_	1.40	159.0290	C_6_H_7_O_5_	0.92	159.0290	C_6_H_7_O_5_	1.21
3							170.1540	C_10_H_20_NO	0.39
4	186.1492	C_10_H_20_NO_2_	1.77						
5				220.2059	C_15_H_26_N	-0.35	220.2058	C_15_H_26_N	−1.03
6	236.2013	C_15_H_26_NO	1.77						
7				238.2168	C_15_H_28_NO	0.96	238.2167	C_15_H_28_NO	0.56
8	254.2118	C_15_H_28_NO_2_	1.44						
9				256.2276	C_15_H_30_NO_2_	1.93	256.2272	C_15_H_30_NO_2_	0.26
10	272.2226	C_15_H_30_NO_3_	0.88						
11	316.3001	C_22_H_38_N	0.83						
12				318.3157	C_22_H_40_N	0.65	318.3158	C_22_H_40_N	0.85
13	334.3106	C_22_H_40_NO	0.59						
14				336.3263	C_22_H_42_NO	0.51	336.3262	C_22_H_42_NO	0.42
15	352.3213	C_22_H_42_NO_2_	0.72						
16				354.3369	C_22_H_44_NO_2_	0.56	354.3369	C_22_H_44_NO_2_	0.56
17	370.3318	C_22_H_44_NO_3_	0.58						
18	492.3330	C_28_H_46_NO_6_	2.18						
19				494.3478	C_28_H_48_NO_6_	0.27	494.3480	C_28_H_48_NO_6_	0.76
20	510.3431	C_28_H_48_NO_7_	1.14						
21				512.3592	C_28_H_50_NO_7_	1.98	512.3593	C_28_H_50_NO_7_	2.10
22	528.3538	C_28_H_50_NO_8_	1.38						
23				530.3693	C_28_H_52_NO_8_	0.98	530.3691	C_28_H_52_NO_8_	0.63
24	546.3630	C_28_H_52_NO_9_	−1.13						
25	668.3648	C_34_H_54_NO_12_	1.04						
26				670.3806	C_34_H_56_NO_12_	1.27	670.3789	C_34_H_56_NO_12_	−1.27
27	686.3731	C_34_H_56_NO_13_	−2.29						
28				688.3909	C_34_H_58_NO_13_	0.87	688.3903	C_34_H_58_NO_13_	−0.01
29	704.3867	C_34_H_58_NO_14_	2.18						
30				706.4016	C_34_H_60_NO_14_	1.02	706.4016	C_34_H_60_NO_14_	1.10
31	722.3966	C_34_H_60_NO_15_	1.11						

It is believed that fragment ions at *m*/*z* values of 200–800 were formed by the cleavage of the tricarballylic acids (TCAs) and the hydroxyl groups from the precursor ions, and these characteristic fragmentations were common to FB1, FB2, and FB3. On the other hand, at *m*/*z* values of 50–200, different fragment ions were thought to form depending on the positions of the hydroxyl groups in compound. In the case of fragment ions from FB1, ID 4 of the 10-carbon chain was formed by the C–C cleavage at C-10, and ID 1 of 2-amino-1-propanol (APA) was formed by the cleavage at C-5. In the case of FB2, the ID 1 of APA was formed by the cleavage at C-5 similar to the fragment ions of FB1, whereas an ion of a 10-carbon chain, like ID 3 and ID 4, was not able to form due to lack of a hydroxyl group at C-10. In contrast, in the case of FB3, ID 1 was not present, owing to lack of a hydroxyl group at C-5, whereas an ID 3 of the 10-carbon chain was formed by the cleavage at C-10. ID 2 was present in all of three product ion spectra, and the calculated formula is C_6_H_7_O_5_, possibly representing TCA.

These results indicate that the fragmentation of FB1, FB2, and FB3 exhibits characteristic patterns, such as the formation of fragment ions by the cleavage of TCAs and the hydroxyl groups from the precursor ion, the formation of different fragment ions depending on the position of the hydroxyl group, and the formation of TCA present in the each compound.

### 2.3. Analysis of Fragment Ions for Compounds I, II, and III

**Figure 4:**
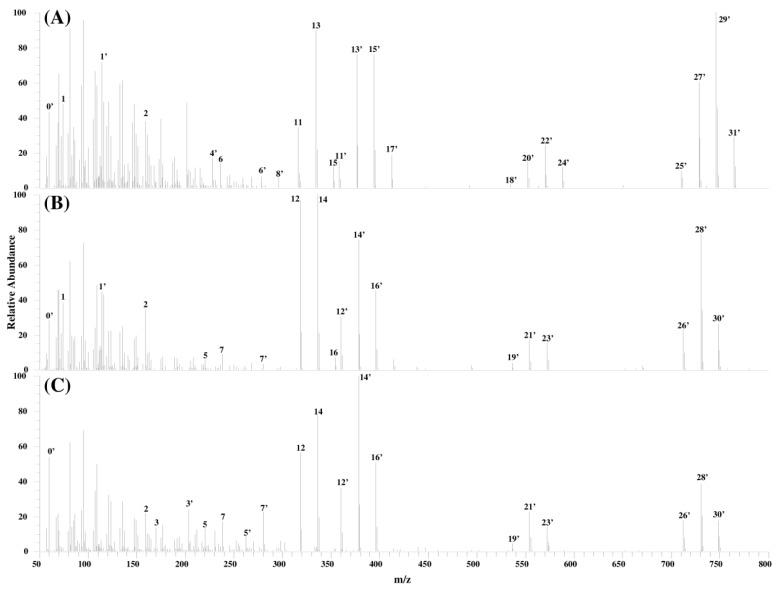
Product ion mass spectra of compounds I, II, and III (**A**) compound I; (**B**) compound II; and (**C**) compound III.

Figure 4 shows the product ion spectra of compounds I, II, and III and Table 3 summarizes the measured mass, the calculated formula, and the mass error for the fragment ions of these compounds.

Compounds I, II, and III shows peaks with the same calculated formulae as that of product ions generated from FB1, FB2, and FB3 in addition to product ions which differed by C_2_H_2_O from the product ions of FB1, FB2, and FB3. Please note that the ions having the same calculated formulae have been denoted with the same ID numbers in Table 3 and the calculated formulae for the ID numbers marked with a dash in Table 3 have an additional C_2_H_2_O.

**Table 3:** Characteristic peak assignment in the product ion mass spectra of compounds I, II, and III.

ID	Compound I			Compound II			Compound III		
	Measured Mass (*m/z*)	Calculated Formula [M + H]^+^	Mass Error (ppm)	Measured Mass (*m*/*z*)	Calculated Formula [M + H]^+^	Mass Error (ppm)	Measured Mass (*m*/*z*)	Calculated Formula [M + H]^+^	Mass Error (ppm)
0'	60.0444	C_2_H_6_NO	0.21	60.0445	C_2_H_6_NO	1.42	60.0444	C_2_H_6_NO	0.72
1	74.0601	C_3_H_8_NO	0.25	74.0601	C_3_H_8_NO	0.97			
1’	116.0706	C_5_H_10_NO_2_	−0.35	116.0707	C_5_H_10_NO_2_	0.70			
2	159.0288	C_6_H_7_O_5_	−0.04	159.0289	C_6_H_7_O_5_	0.53	159.0289	C_6_H_7_O_5_	0.44
3							170.1541	C_10_H_20_NO	0.75
3'							212.1646	C_12_H_22_NO_2_	0.21
4'	228.1594	C_12_H_22_NO_3_	−0.13						
5				220.2060	C_15_H_26_N	0.29	220.2061	C_15_H_26_N	0.42
5'							262.2168	C_17_H_28_NO	0.99
6	236.2003	C_15_H_26_NO	−2.38						
6'	278.2114	C_17_H_28_NO_2_	−0.20						
7				238.2167	C_15_H_28_NO	0.84	238.2164	C_15_H_28_NO	−0.78
7'				280.2264	C_17_H_30_NO_2_	−2.70	280.2274	C_17_H_30_NO_2_	0.89
8'	296.2205	C_17_H_30_NO_3_	−2.07						
11	316.2997	C_22_H_38_N	−0.61						
11'	358.3106	C_24_H_40_NO	0.39						
12				318.3156	C_22_H_40_N	0.08	318.3155	C_22_H_40_N	−0.02
12'				360.3261	C_24_H_42_NO	−0.12	360.3260	C_24_H_42_NO	−0.37
13	334.3102	C_22_H_40_NO	−0.78						
13'	376.3208	C_24_H_42_NO_2_	−0.46						
14				336.3261	C_22_H_42_NO	0.15	336.3260	C_22_H_42_NO	−0.21
14'				378.3367	C_24_H_44_NO_2_	0.12	378.3367	C_24_H_44_NO_2_	0.12
15	352.3202	C_22_H_42_NO_2_	−2.40						
15'	394.3315	C_24_H_44_NO_3_	−0.07						
16				354.3371	C_22_H4_4_NO_2_	1.34			
16'				396.3475	C_24_H_46_NO_3_	0.64	396.3473	C_24_H_46_NO_3_	0.25
17'	412.3418	C_24_H_46_NO_4_	−0.75						
18'	534.3431	C_30_H_48_NO_7_	1.14						
19'				536.3582	C_30_H_50_NO_7_	0.08	536.3591	C_30_H_50_NO_7_	1.66
20'	552.3516	C_30_H_50_NO_8_	−2.67						
21'				554.3691	C_30_H_52_NO_8_	0.72	554.3691	C_30_H_52_NO_8_	0.61
22'	570.3637	C_30_H_52_NO_9_	−0.02						
23'				572.3793	C_30_H_54_NO_9_	−0.06	572.3794	C_30_H_54_NO_9_	0.15
24'	588.3752	C_30_H_54_NO_10_	1.63						
25'	710.3760	C_36_H_56_NO_13_	2.00						
26'				712.3914	C_36_H_58_NO_13_	1.53	712.3902	C_36_H_58_NO_13_	−0.10
27'	728.3850	C_36_H_58_NO_14_	−0.32						
28'				730.4010	C_36_H_60_NO_14_	0.23	730.4014	C_36_H_60_NO_14_	0.82
29'	746.3956	C_36_H_60_NO_15_	−0.24						
30'				748.4123	C_36_H_62_NO_15_	1.20	748.4118	C_36_H_62_NO_15_	0.54
31'	764.4059	C_36_H_62_NO_16_	−0.48						

For *m*/*z* values of 700–800, IDs 25'–31' were observed at equal intervals. Since the difference between the calculated formulae suggested the presence of the hydroxyl groups, it was concluded that compound I had three hydroxyl groups, and that compounds II and III had two hydroxyl groups each. The same results were obtained for *m*/*z* values of 500–600 (ID 18'–24').

At *m*/*z* values of 300–450, the same peaks (IDs 11–16) of product ions generated from FB1, FB2, and FB3 were observed in addition to the peaks corresponding to FB1, FB2, and FB3 with an additional C_2_H_2_O (ID 11'–17'). It was assumed that the cleavage of C_2_H_2_O in compounds I, II, and III produced the same fragment ions as FB1, FB2, and FB3. This assumption was also made at *m*/*z* values of 50–300 where IDs 1–7, as well as IDs 1'–7', generated by the cleavage of C_2_H_2_O were observed. In addition, ID 2, which was a product ion common to FB1, FB2, and FB3, was also observed in the case of compounds I, II, and III. Considering that ID 2 represented TCA in the product ion mass spectra of FB1, FB2, and FB3, it was presumed that TCA is also bound to compounds I, II, and III. Further, different fragment ions depending on the positions of the hydroxyl groups were observed in the spectra of compounds I, II, and III. This is characteristic of the fragmentation in FBs. Spectra for compound I showed the existence of ID 4' and ID 1' formed presumably by the cleavage at C-10 and C-5, respectively. Compound II showed the presence of ID 1' and the absence of spectra like ID 3' and 4', whereas compound III showed the existence of ID 3' and the absence of ID 1'. From these observations, it was assumed the hydroxyl groups are bound to compound I at C-5 and C-10, to compound II at C-10, and to compound III at C-5 positions.

It was hypothesized that compounds I, II, and III have TCAs, hydroxyl groups, and C_2_H_2_O and that the fragmentation of compounds I, II, and III are similar those of FB1, FB2, and FB3, respectively. Since compounds I, II, and III supposedly have similar structures to FBs, with an additional C_2_H_2_O in their structures, it was suggested that these compounds are FA1, FA2, and FA3, which are *N*-acetyl analogs of FB1, FB2, and FB3, respectively. In order to confirm the above hypothesis, we attempted to synthesize FA1 from the FB1 standard material, which is easily obtained, and compared the product ions of compound I with those of the synthesized FA1.

### 2.4. Characterization of Compound I Using the FA1 Standard

FA1, synthesized from the FB1 standard material, was analyzed using the LC-Orbitrap MS. The measured mass, the calculated formulae, the theoretical mass, and the mass error were 764.4087, C_36_H_62_NO_16_^+^, 764.4063, and 3.11 ppm, respectively.

The NMR spectral measurements of the synthesized product indicated a chemical shift of the proton at C-2 at ~3.9 ppm. Since the chemical shift of the proton at C-2 was found to be ~3.1 ppm in the case of FB1, this result further validated that the synthesized compound is an *N*-acetyl analog of FB1.The chemical shifts (δ) for the other protons in the ^1^H NMR (methanol-d_4_) measurements were 1.002 (t, *J* = 0.012 Hz, 3H), 1.025–1.100 (m, 6H), 1.235 (d, *J* = 0.012 Hz, 3H), 1.323–1.632 (m, 18H), 1.690–1.852 (m, 2H), 1.917 (brs, 1H), 2.052 (s, 3H), 2.573–2.944 (m, 8H), 3.254–3.335 (m, 2H), 3.719 (brs, 1H), 3.852–3.909 (m, 2H), 3.957–4.020 (m, 1H), 5.069 (dd, *J* = 0.005, 0.014 Hz, 1H), and 5.259 (td, *J* = 0.005, 0.018 Hz, 1H). These results agreed with the values we observed for FB1 and those seen in previous studies [7,20]. Based on the findings, the synthesized compound was identified as FA1. The purity of the synthesized FA1 was determined to be 87.0%.

**Figure 5:**
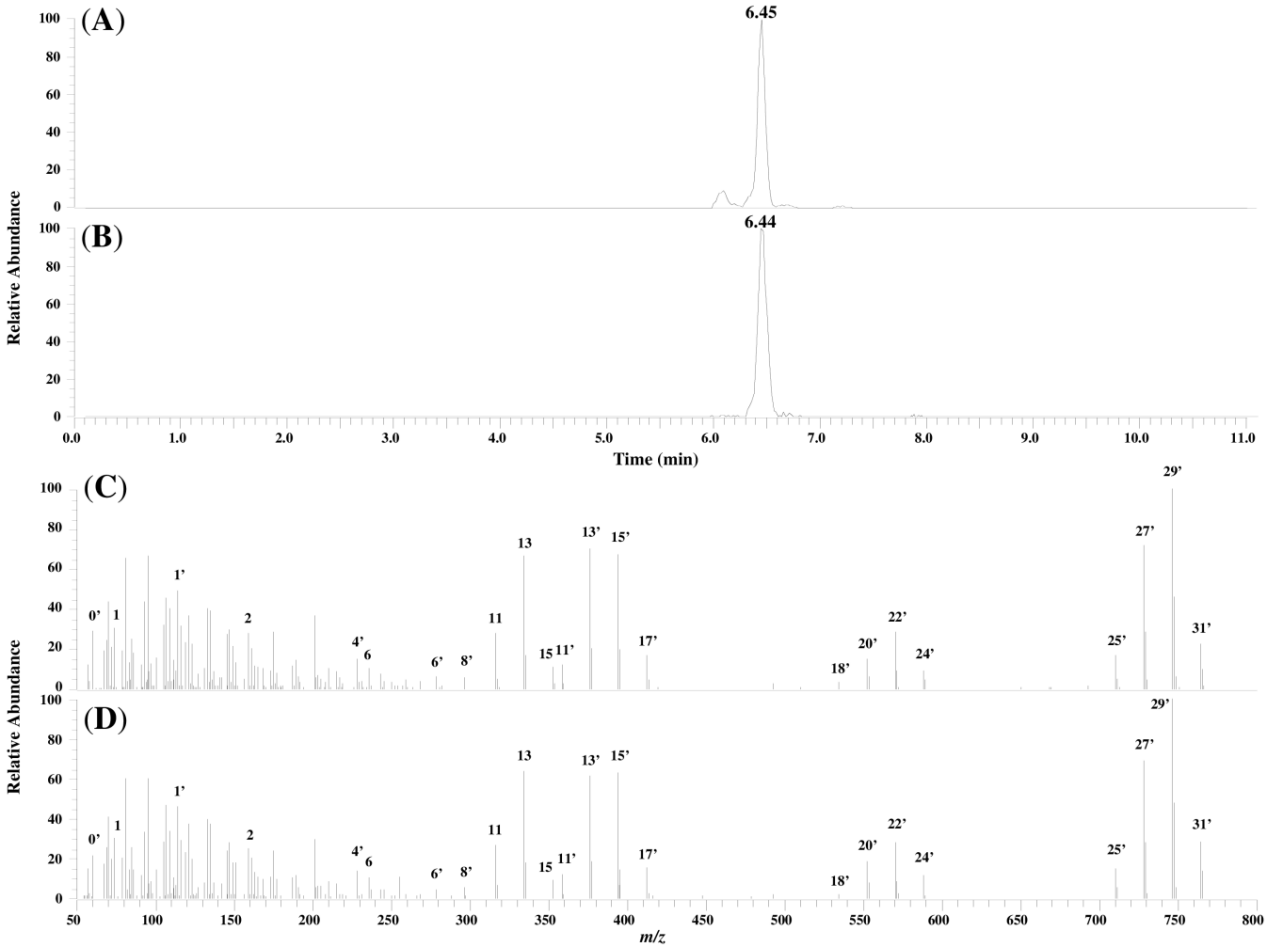
Chromatograms and product ion mass spectra of compound I and synthesized FA1: (**A**) chromatogram of compound I in the sample; (**B**) chromatogram of synthesized FA1; (**C**) product ion mass spectrum of compound I in the sample; and (**D**) product ion mass spectrum of synthesized FA1.

Figure 5 compares the chromatograms and product ion mass spectra of compound I in the corn sample and the synthesized FA1 standard. The similarity in their retention times and the profile of the product ion mass spectra lent further evidence that compound I was FA1.

The main fragment ions generated from FA1 were identified from the product ion spectra. ID 0', ID 1', and ID 2 were assigned to *N*-acetyl, 2-acetamide-1-propanol (the fragment ion formed by the cleavage of the hydroxyl group at C-5), and TCA, respectively. IDs 3'–4' and IDs 11'–13' were assigned to the fragment ions generated by the cleavage of the hydroxyl group at C-10 and those generated by the cleavage of TCA and the hydroxyl groups, respectively. Considering the evidence in Section 2, along with the above results, it was probably indicated that compounds II and III are FA2 and FA3, respectively.

### 2.5. Determination of FA1, FB1, FB2, and FB3 in MTC-9999E

Next, the accuracy of the method for the determination of FA1, FB1, FB2, and FB3 was evaluated, and the amounts of FA1, FB1, FB2, and FB3 in the MTC-9999E sample were quantified. The results are shown in Table 4.

**Table 4:** The performance of the method and the amount of fumonisins in MTC-9999E.

Validation item	FA1	FB1	FB2	FB3
Linearity (*r*)	0.9987	0.9989	0.9995	0.9972
Recovery (%)	101.8	99.0	100.2	96.4
Intraday-precision (%)	5.3	9.0	5.0	10.8
LOD (μg/kg)	0.73	0.07	0.15	0.12
LOQ (μg/kg)	2.44	0.20	0.49	0.40
Analytical level (mg/kg)	4.2	28.6	8.9	2.0
Acceptance limit (mg/kg)	-	28.3 ± 7.6	7.1 ± 1.9	1.7 ± 0.5

The coefficient of linearity, the recovery, the intraday precision, and the LODs were determined to be >0.99, 96.4%–101.8%, 5.0%–10.8%, and 0.07–0.73 μg/kg, respectively. Thus, it can be concluded that we have developed a well performing method for the simultaneous analysis of FA1, FB1, FB2, and FB3. The method was applied to the analysis of MTC-9999E. Since the individual concentrations of FB1 and FB2 in MTC-9999E were already known and were over the range of each of the calibration curves, the sample was diluted 10 times prior to analysis. The analytical levels of FB1, FB2, and FB3 in MTC-9999E were within the acceptance limits, which reconfirmed the validity of the method. The amount of FA1 in MTC-9999E was found to be 4.2 mg/kg, which was approximately 15% of the amount of FB1. These findings confirm the presence of FAs in addition to FBs in corn, as potential food contaminants.

## 3. Experimental Section

### 3.1. Sample, Chemicals, and Reagents

Mycotoxin reference material (MTC-9999E) obtained from Trilogy Analytical laboratory (Washington, DC, USA) was used as the corn sample naturally contaminated with fumonisins. The acceptance limits incorporating 26.8% of uncertainty are 28.3 ± 7.6 mg/kg of FB1, 7.1 ± 1.9 mg/kg of FB2, and 1.7 ± 0.5 mg/kg of FB3, respectively.

FB1 standard was obtained from Cayman Chemical Co. (Ann Arbor, MI, USA). Standard solutions containing 50 μg/mL of FB1, FB2, and FB3 in acetonitrile/water (1/1 *v*/*v*) were from Romer Labs (Bukit Merah, Singapore). Methanol (LC/MS grade), acetonitrile (analytical grade), acetic acid (guaranteed reagent grade), ammonium acetate (analytical grade), dipotassium hydrogen phosphate (guaranteed reagent grade), *N*,*N*-dimethylformamide (guaranteed reagent grade), and acetic anhydride (guaranteed reagent grade) were purchased from Kanto Chemical Co., Inc. (Tokyo, Japan). Methanol-d_4_ (NMR grade) and Supelpak 2 were obtained from Merck KGaA (Darmstadt, Germany) and Sigma-Aldrich (Bellefonte, PA, USA), respectively. Water was purified using a Millipore (Molsheim, France) Milli-Q system. Q-sep Q 110 used as a QuEChERS extraction kit was obtained from RESTEK (Bellefonte, PA, USA). MultiSep 229 Ochra used as a multi-functional cartridge was obtained from Romer Labs (Bukit Merah, Singapore). A PTFE filter with a mesh size of 0.20 μm was procured from Advantec Toyo Kaisha, Ltd. (Tokyo, Japan).

### 3.2. Sample Preparation

Sample preparation was carried out as previously described [21]. Namely, a 2.5 g sample was placed in a 50 mL polypropylene centrifuge tube and 20 mL of a 2% acetic acid aqueous solution/acetonitrile (1:1 *v*/*v*) was added to the sample. They were mixed at 250 rpm using a shaker (SR-2 DS; Taitec Saitama, Japan) for 1 h. The contents of the Q-sep Q110 were then added to the tube. The mixture was vortexed for 20 s and centrifuged at 3000 rpm for 5 min. The supernatant (acetonitrile phase) was frozen at −30 °C for 1 h and was again centrifuged at 3000 rpm for 5 min. Following this, 5 mL of the supernatant, 1 mL of water, and 60 μL of acetic acid were mixed together, and the mixture was applied to a MultiSep 229 Ochra. The eluate (4 mL) was dried at 40 °C under a nitrogen stream and dissolved in 400 μL of a 10 mM ammonium acetate aqueous solution/acetonitrile (85:15 *v*/*v*). Each sample was filtered with a 0.20 μm PTFE filter immediately before LC-Orbitrap MS analysis.

### 3.3. LC-Orbitrap MS Analysis

The LC-Orbitrap MS analysis was performed using an Ultimate 3000 system coupled to a Q-Exactive™ mass spectrometer (Thermo Fisher Scientific, Bremen, Germany). The Xcalibur™ 2.2 software (Thermo Fisher Scientific, Bremen, Germany) was used to control the instruments and to process the data.

LC was performed by using a 10 mM ammonium acetate aqueous solution as solvent A and 2% acetic acid in methanol as solvent B [21]. The gradient profile used was 2% B (0–2.0 min), 55% B (3.0–4.0 min), 70% B (4.1 min), 80% B (7.0 min), 95% B (7.01–8.0 min), and 2% B (8.01–11.0 min). The flow rate was set to 0.4 mL/min and the column temperature was maintained at 40 °C. The chromatographic separation was carried out on a Mastro C18 column (2.1 mm × 100 mm, 3 μm) obtained from Shimadzu GLC (Tokyo, Japan) using an injection volume of 5 μL.

The Q-Exactive™ mass spectrometer was operated in positive mode with a heated electrospray ionization source (HESI-II) and a spray voltage of 3.00 kV. The capillary and the heater temperatures were 350 °C and 300 °C, respectively. The sheath gas and the auxiliary gas flow rates were applied 40 and 10 (in arbitrary units), respectively. The precursor ion scan was done in the full MS mode at a resolution of 70,000 at an *m*/*z* value of 200 (3 scans/s), auto gain control (AGC) target of 3e6, maximum injection time (IT) of 100 ms, and a scan range of *m*/*z* values of 100–1000. The product ion scan was conducted in a data dependent MS^2^ mode (dd-MS^2^) using a resolution of 17,500 at a *m*/*z* value of 200, AGC target of 2e5, maximum IT of 200 ms, normalized collision energy (NCE) of 30 eV, stepped NCE of 50%, and a scan range of *m*/*z* 50–800.

### 3.4. Synthesis of FA1 from FB1 and the Characterization of the Structures by NMR Analysis

FA1 was synthesized from FB1 as follows [22,23]. FB1 weighed 4.61 mg in a 50 mL recovery flask and was dissolved in 0.2 mL of *N*,*N*-dimethylformamide. Following this, 1.5 mL of 3 M dipotassium hydrogen phosphate aqueous solution and 1.5 mL of acetic anhydride were added to the FB1 solution and stirred with a magnetic stirrer for 10 min. To the reaction mixture, 3 mL of water was added, and the solution was further stirred for 30 min. To this solution, 50 mL of water was added and the full volume of the reaction solution was loaded on the Supelpak 2 previously filled in an open column. The column loaded with the reaction solution was washed five times with 15 mL of water and once with 10 mL of 50% acetonitrile solution. The compounds were then extracted with 60 mL of 50% acetonitrile solution and the extract was evaporated to obtain 2.69 mg of FA1. A portion of the FA1 was dissolved again with methanol-d_4_ and analyzed by NMR spectroscopy.

^1^H NMR (600 MHz) and COSY spectra were recorded on a Bruker AV 600 instrument (Bruker, Karlsruhe, Germany), and the chemical shifts are expressed in terms of δ (ppm) relative to the solvent signal (methanol-d_4_, δ^H^ 3.31, δ^C^ 49.0).

### 3.5. Validation of the Method

The method was validated by evaluating the linearity, recovery, and the intraday precision using a corn grit sample which contained 9.3 μg/kg of FB1. (FB2, FB3, and FA1 were not detected.). The coefficient of linearity was determined from the calibration curves, which were constructed by plotting the peak areas of the prepared samples spiked with FA1, FB1, FB2, and FB3 standards against the concentrations of the analyte. The concentrations of FA1, FB1, FB2, and FB3 added to the samples were 5, 10, 50, 100, 500, 1000, and 5000 μg/kg. To the sample, 50 μg/kg of FA1, FB1, FB2, and FB3 were added for recovery and intraday precision evaluations. The intraday precision was calculated by repeating the measurements five times on the same day and calculating the relative standard deviation of these measurements. The LODs and the LOQs were defined as the concentrations that gave a signal to noise ratio (S/N) of 3:1 and 10:1, respectively.

## 4. Conclusions

Three peaks of compounds I, II, and III (presumably representing FAs), as well as FBs were detected simultaneously from the corn sample naturally contaminated with fumonisins (MTC-9999E) using high-resolution LC-Orbitrap MS. In the case of FB1, FB2, and FB3, similar fragment ions generated by the cleavage of TCAs and the hydroxyl groups were observed at *m*/*z* values of 200–800, and different ions (depending on the bonding position of the hydroxyl group in each compound) were detected at *m*/*z* values of 50–200. From the results of the fragment ions analysis for the compounds, it was hypothesized that compounds I, II, and III have similar structures as FBs with the inclusion of an additional C_2_H_2_O bound to the compound. Further, compound I has the same retention time and product ion mass spectrum as those of FA1 synthesized from FB1 standard material. Based on these results, compound I was identified as FA1 and consequently, compounds II and III were hypothesized to be FA2 and FA3, respectively.

A method for determining of FA1, FB1, FB2, and FB3 was developed and the validated method was applied to the analysis of the MTC-9999E sample. The analytical levels calculated for FB1, FB2, and FB3 were within the acceptance limits, which reconfirmed the validity of the method. The amount of FA1 in MTC-9999E was determined to be 4.2 mg/kg, which was approximately 15% of the amount of FB1.

These findings using high-resolution LC-Orbitrap MS indicate that corn may contain not only FBs but also FAs, as potential contaminants. Our method can be applied to control the occurrence of fumonisins in corn products.
